# Nitrosative and Oxidative Stresses Contribute to Post-Ischemic Liver Injury Following Severe Hemorrhagic Shock: The Role of Hypoxemic Resuscitation

**DOI:** 10.1371/journal.pone.0032968

**Published:** 2012-03-05

**Authors:** Emmanuel E. Douzinas, Olga Livaditi, Marios-Konstantinos Tasoulis, Panagiotis Prigouris, Dimitrios Bakos, Nikolaos Goutas, Dimitrios Vlachodimitropoulos, Ilias Andrianakis, Alex Betrosian, George D. Tsoukalas

**Affiliations:** 1 3rd Department of Critical Care Medicine, University of Athens Medical School, Athens, Greece; 2 Department of Forensic Medicine & Toxicology, University of Athens Medical School, Athens, Greece; 3 4th Pulmonary Clinic, Sotiria Respiratory Hospital, Athens, Greece; Rutgers University, United States of America

## Abstract

**Purpose:**

Hemorrhagic shock and resuscitation is frequently associated with liver ischemia-reperfusion injury. The aim of the study was to investigate whether hypoxemic resuscitation attenuates liver injury.

**Methods:**

Anesthetized, mechanically ventilated New Zealand white rabbits were exsanguinated to a mean arterial pressure of 30 mmHg for 60 minutes. Resuscitation under normoxemia (Normox-Res group, n = 16, PaO_2_ = 95–105 mmHg) or hypoxemia (Hypox-Res group, n = 15, PaO_2_ = 35–40 mmHg) followed, modifying the FiO_2_. Animals not subjected to shock constituted the sham group (n = 11, PaO_2_ = 95–105 mmHg). Indices of the inflammatory, oxidative and nitrosative response were measured and histopathological and immunohistochemical studies of the liver were performed.

**Results:**

Normox-Res group animals exhibited increased serum alanine aminotransferase, tumor necrosis factor - alpha, interleukin (IL) -1β and IL-6 levels compared with Hypox-Res and sham groups. Reactive oxygen species generation, malondialdehyde formation and myeloperoxidase activity were all elevated in Normox-Res rabbits compared with Hypox-Res and sham groups. Similarly, endothelial NO synthase and inducible NO synthase mRNA expression was up-regulated and nitrotyrosine immunostaining increased in animals resuscitated normoxemically, indicating a more intense nitrosative stress. Hypox-Res animals demonstrated a less prominent histopathologic injury which was similar to sham animals.

**Conclusions:**

Hypoxemic resuscitation prevents liver reperfusion injury through attenuation of the inflammatory response and oxidative and nitrosative stresses.

## Introduction

Hemorrhagic shock and resuscitation initiates an inflammatory response characterized by the up-regulation of cytokine expression and accumulation of neutrophils in a variety of tissues [1], [2]. Liver with its crucial involvement in metabolism and homeostasis is among the most frequently affected organs [3]. These processes are triggered when liver is transiently deprived of oxygen and re-oxygenated. This occurs in a number of clinical settings associated with low flow states resulting in insufficient perfusion, such as hemorrhagic and other types of shock, diverse surgical procedures, or during the organ procurement for transplantation [4], [5].

Although ischemia causes significant injury to tissues and cells, the injury during reperfusion is more severe [5]. Animal studies have shown that early in the reperfusion period, tissue damage appears to be associated with a decreased amount of endothelial nitric oxide (NO) synthase (e-NOS) derived NO related to e-NOS down-regulation [6]. In contrast inducible NO synthase (i-NOS) derived NO is produced in excessive amounts related to i-NOS up-regulation after hemorrhage [7], [8]. Similarly, reactive oxygen species (ROS) have been shown to exert a central role in contributing to tissue injury after reperfusion of the ischemic liver [9]. The rapid interaction of ROS, superoxide in particular, with the iNOS derived NO, produces peroxynitrite radical [10] that seems to denature DNA, inhibit phosphorylation, and cause lipid peroxidation [10]. Peroxynitrite, among others [myeloperoxidase (MPO)], may nitrate proteins resulting in nitrotyrosine, the detection of which represents a reliable marker of tissue damage [11]. This knowledge is further supported by the beneficial effect exerted either from radical scavengers (N-acetylcysteine, superoxide dismutase or catalase) [12], [13], or by selective iNOS inhibitors [N6-(iminoethyl)-L-lysine or N3-(aminomethyl) benzylacetamidine] [7], [8], that all offer a varying degree of protecting promise in liver reperfusion injury.

While hypoxia leads to an accumulation of reducing equivalents, no longer able to be oxidized by mitochondria due to limited oxygen, the sudden rise in oxygen at the onset of reperfusion is considered to lead to oxidative stress [4], [14]. The ensuing oxidative aggression may lead to hepatocyte damage [15], [16] contributing to the development of hypoxic hepatitis [16].

We have shown the beneficial effect of the gradual re-introduction of O_2_ to the ischemic tissues during resuscitation from hemorrhagic shock by means of “hypoxemic resuscitation” [17], [18], [19]. These effects have been observed in both, the organ tissues [18], [19] and the systemic interactions [17]. The aim of the present study therefore, was to investigate the effect of hypoxemic resuscitation from hemorrhagic shock in the prevention of the referred type of liver ischemia reperfusion injury. This effect was assessed by the degree of oxidative, nitrosative and inflammatory responses afforded in the livers of animals subjected to hemorrhagic shock and resuscitation.

## Results

### Serum alanine aminotransferase activity

**Figure 1:**
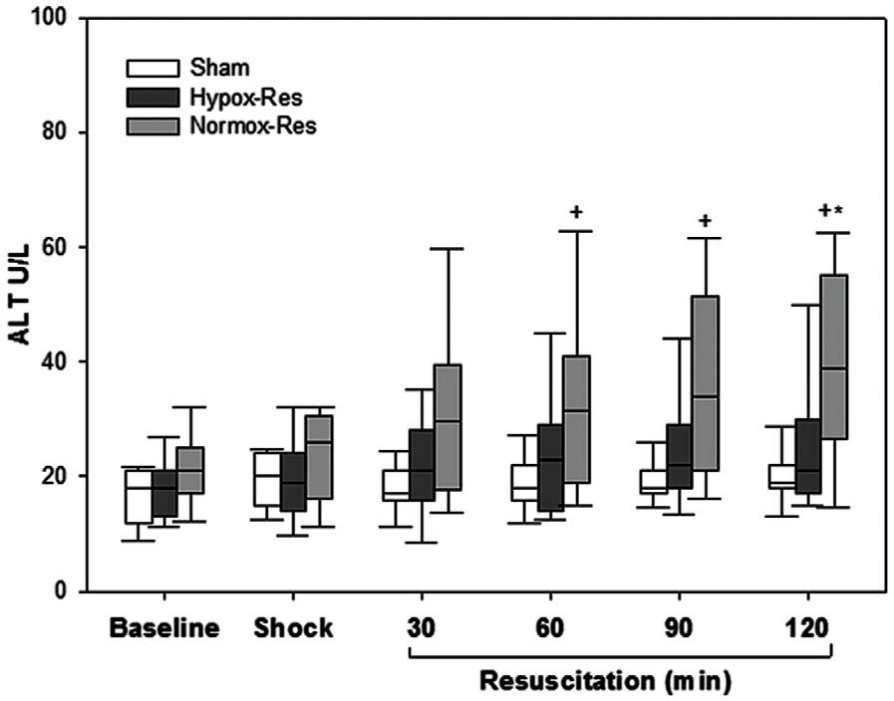
Serum levels of alanine aminotransferase (ALT) denoting the degree of hepatocellular injury. Box plots show the median (*lines*), interquartile ranges (*boxes*) and 5 and 95 percentiles (*whiskers*) of the three groups during the experiment. ^+^ *p*<0.05 Sham vs. Normox-Res; *p<0.05 Normox-Res vs. Hypox-Res.

As shown in Figure 1, serum alanine aminotransferase (ALT) activities increased significantly at 60 min of reperfusion and thereafter in Normox-Res group compared with sham. By contrast, ALT activity showed no difference at all time points of reperfusion between Hypox-Res and sham groups. Yet, significant difference was observed between the Normox-Res and Hypox-Res at 120 min of reperfusion.

### Hepatic oxidative stress

**Figure 2:**
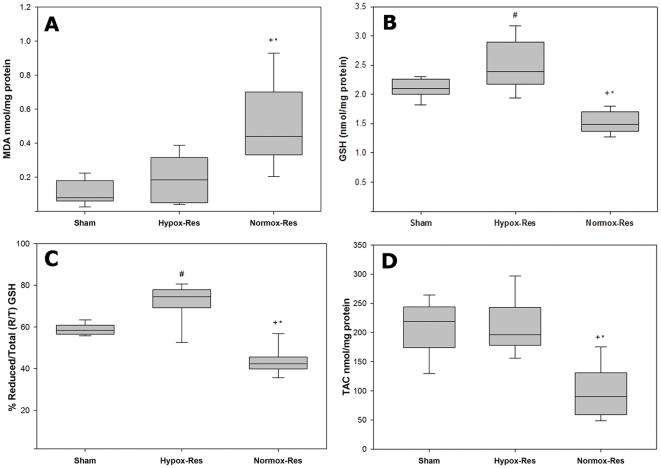
Indices of oxidative stress. Hepatic tissue levels of malondialdehyde (*MDA, panel A*), glutathione (*GSH, panel B*), the percentile ratio of reduced to total GSH (*R/T, panel C*) and the geometric mean fluorescence intensities of neutrophils and monocytes of peripheral blood which reflect reactive oxygen species (ROS) production (*GMFI, panel D*). Box plots showing the median (*lines*), interquartile ranges (*boxes*), and the 5 and 95 percentiles (*whiskers*) of the three groups at the end of the experiment. ^+^ *p*<0.05 Sham vs. Normox-Res; *p<0.05 Normox-Res vs. Hypox-Res.

Reperfusion injury in Normox-Res treated animals led to increased hepatic tissue malondialdehyde (MDA) levels, indicating lipid peroxidation, compared with both sham and Hypox-Res groups (p<0.05, Figure 2A). By contrast, MDA levels of Hypox-Res group were similar to sham group (p = NS). Also, ischemia/reperfusion (I/R) injury caused significant exhaustion of the hepatic antioxidant defense in Normox-Res group as observed by the significant reduction in the reduced glutathione (GSH) of hepatic tissue compared with both Hypox-Res and sham groups (p<0.05, Figure 2B) in which it was maintained. Hepatic tissue GSH-to-total GSH (GSH+2 oxidized, GSSG) ratio (Figure 2C) was significantly lower in Normox-Res group compared with both Hypox-Res and sham groups (p<0.05).

The generation of ROS was enhanced in Normox-Res treated animals compared with both Hypox-Res and sham. As shown in Figure 2D the median geometric mean of fluorescence intensity (GMFI), representing ROS formation, in the Normox-Res group was significantly higher than Hypox-Res and sham groups (p<0.05). In contrast, no significant differences were observed between Hypox-Res and sham groups.

### Hepatic myeloperoxidase activity

As shown in Table 1, I/R injury caused a significant increase in hepatic neutrophil infiltration in the Normox-Res group, assessed by elevated hepatic myeloperoxidase (MPO) activity, compared with Hypox-Res and sham groups (p = 0.05). MPO activity was also significantly elevated between Hypox-Res and sham groups (p = 0.05).

**Table 1:** Myeloperoxidase activity and eNOS expression.

	Sham	Hypox-Res	Normox-Res
MPO Activity/g tissue/min	8.9 (7.7–12.9)	18.6 (13.2–36.4)#	40.3 (26.5–56.4)+,*
e-NOS expression by the [2^−ΔΔCT^] method	0.11 (0.04–0.17)	0.3 (0.2–0.34)	0.45 (0.18–0.6)+,*

Median liver tissue myeloperoxidase (MPO) activity and endothelial NO synthase (e-NOS) expression at 120 min of reperfusion in all three groups (*parentheses*, interquartile range).

+ p<0.05 Sham vs. Normox-Res groups.

* p<0.05 Hypox-Res vs. Normox-Res groups.

# p<0.05 Sham vs. Hypox-Res.

### RT-PCR for e-NOS and i-NOS mRNA expression

The expression of e-NOS mRNA in liver tissue of the Normox-Res group animals was significantly increased compared with Hypox-Res and sham groups (Table 1, p<0.05). A borderline association of e-NOS mRNA was showed between Hypox-Res and sham groups (p = 0.056). The expression of i-NOS mRNA was detected in the liver tissue of 12 out of 16 animals of the Normox-Res group vs. of 3 out of 15 animals in the Hypox-Res group (p<0.05). In contrast, i-NOS mRNA expression was not detected in the liver tissue of the sham group showing no difference to Hypox-Res group.

### Serum cytokines

**Figure 3:**
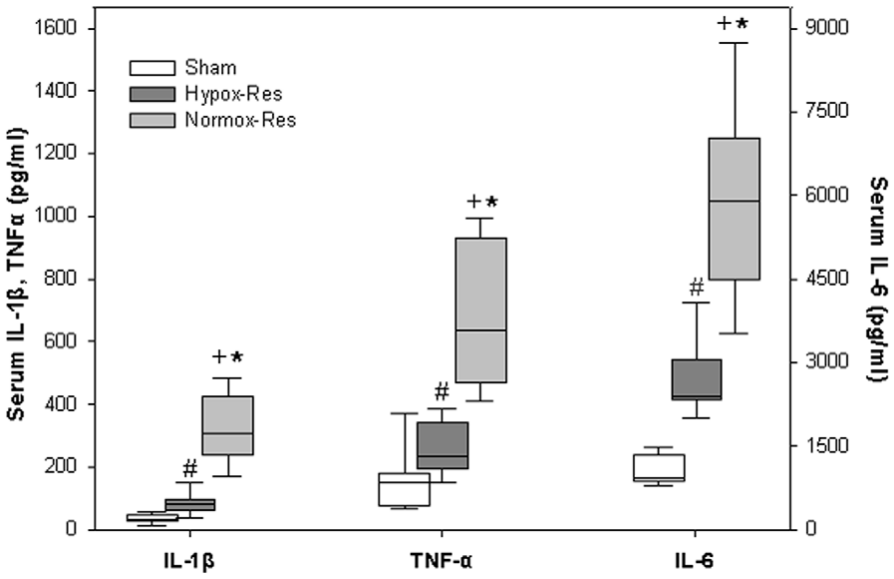
Serum cytokines. Serum levels of Tumor Necrosis Factor (TNF) – α, Interleukin (IL) - 1β and IL-6. Box plots showing the median (*lines*), interquartile ranges (*boxes*) and 5 and 95 percentiles (*whiskers*) of the three groups at the end of the experiment. ^+^ *p*<0.05 Sham vs. Normox-Res; *p<0.05 Normox-Res vs. Hypox-Res; # p<0.05 Sham vs. Hypox-Res group.

Serum Tumor Necrosis Factor-alpha (TNF-α), Interleukin (IL) -1β and IL-6 levels are shown in Figure 3. It appears that the levels of all cytokines of Normox-Res group are significantly higher than those of Hypox-Res group (p<0.05). Likewise, the levels of all cytokines of Hypox-Res group are significantly higher than those of sham group (p<0.05).

### Morphological assessment

**Figure 4:**
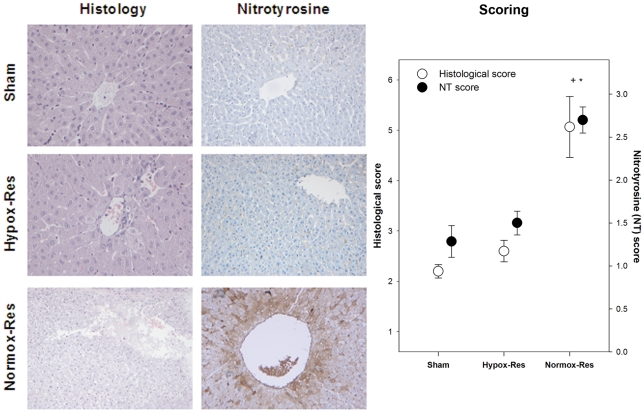
Histopathological and immunohistochemical morphological changes. Slices for histology (left column) were stained with hematoxylin/eosin, while for nitrotyrosine (NT) immunohistochemical detection (right column) were stained with the chromogene 3,3 diaminobenzidine tetrahydrochloride. As shown, the central vein originated from a Normox-Res animal is completely destructed and the adjacent hepatocytes have been intensively stained for NT. In contrast, in the case of hypoxemic animals the respective region is well preserved with limited staining for NT, an appearance similar to that of sham animals. These differences are shown in the box plot in where the median (*lines*) histological and NT scores, interquartile ranges (*boxes*), and 5 and 95 percentiles (*whiskers*) of the three groups at the end of the experiment are presented. ^+^ *p*<0.05 Sham vs. Normox-Res; *p<0.05 Normox-Res vs. Hypox-Res.

In Figure 4 left column (Histology), the common histology of zone III, is presented. Degenerative changes of the central vein are shown with necrosis of adjacent hepatocytes, a representative image of the Normox-Res animals. In contrast, the respective area in Hypox-Res animals is well preserved and appears to be similar to sham animals.

In the middle column (Nitrotyrosine) of Figure 4, immunohistostaining reveals dense nitrotyrosine staining around the central vein in Normox-Res animals. The central vein appears degenerated and dilated representing a common finding in the slides of this group. In contrast, minimal degree of nitrotyrosine staining appears around the well preserved central vein of Hypox-Res slices, a finding that resembles to those of sham animals.

The histological and nitrotyrosine scores are presented in Figure 4 right column (Scoring). Histological analysis of the sham group revealed normal architecture with a low injury score (mean+SE Suzuki score 2.20±0.13). The sections of livers obtained from the Hypox-Res group showed similar morphology to those of sham animals. Namely, a significant preservation of the lobular architecture was appeared with no signs of hepatocyte necrosis, despite the slightly congested sinusoids (mean+SE Suzuki score 2.60±0.21, p = NS compared with sham group). In contrast, biopsies from Normox-Res group demonstrated significant evidence of injury with severe sinusoidal/vascular congestion and marked vacuolization focally associated with minimal hepatocyte necrosis (mean±SE Suzuki score of 5.06±0.60, p<0.05 compared with sham and Hypox-Res groups). Mild neutrophil infiltration in the lobule was observed in biopsies obtained from both shocked groups.

Hepatic mean±SEM nitrotyrosine score, was significantly increased in Normox-Res (2.7±0.15) compared with both Hypox-Res (1.5±0.14, p<0.05) and sham (1.29±1.18, p<0.05) groups and is also presented in Figure 4, right column.

## Discussion

The present study was to verify the protective effect of hypoxemic reperfusion on post-ischemic liver injury in an experimental model of severe hemorrhagic shock and resuscitation.

In the absence of any protective antioxidant therapy, the results of this study showed that the gradual reintroduction of oxygen during the initial reperfusion period attenuates post-ischemic liver injury by limiting both oxidative and nitrosative stresses, as well as the inflammatory response. Animals resuscitated under normoxemia had severe liver damage associated with increased serum ALT levels, increased MDA formation and hepatic MPO activity, up-regulation of e-NOS and i-NOS mRNA expression and augmented nitrotyrosine formation. Both whole blood ROS activity and serum proinflammatory cytokines were also elevated. Hypoxemic resuscitation alleviated these findings and attenuated hepatic tissue injury.

It seems paradox however, how an already stressed animal under exsanguinated shock may afford hypoxemia at the initial reperfusion period. Dealing with this observation it should be argued that at the onset of resuscitation, shock reverts by the transfusion of shed blood while tissues have been adapted at a low O_2_ state; at this precise instant, excess of O_2_ re-entry should be prevented. Consequently, we claim that as the insult (hemorrhagic shock) subsides by transfusion, oxygen supply should be low and gradually should increase at quantities corresponding to the increasing tissue requirements. In the clinical setting, this is reliably reflected by the changes of mixed venous blood O_2_ saturation (SvO_2_). In fact, hypoxemic reperfusion in a porcine model of intestinal ischemia was associated with a trend towards gradual restoration of SvO_2_ in contrast to normoxemia in which after a brief increase, SvO_2_ declined abruptly reflecting apparently hemodynamic deterioration [20]. The progressive O_2_ re-entry to ischemic tissues is the background hypothesis of the current study.

Regarding hepatic ischemia, it initially activates Kupffer cells, which are the main source of vascular reactive oxygen formation during the initial reperfusion period [21]. Apparently, since ROS in the current study were measured in the peripheral blood, no direct data for hepatic oxidative molecules production is provided. However, the ensuing generalized oxidative stress that overwhelmed the antioxidant ability of liver reflects the link between circulating ROS and the liver-victim of oxidant aggression. This association was revealed by the enhanced lipid peroxidation detected by the increased levels of MDA and the consumption of GSH in the liver tissue of normoxemically resuscitated animals. Namely, during resuscitation, increased GMFI detection in the blood of Normox-Res animals denotes massive ROS production [22]. Especially, at the onset of reoxygenation, free radical production can overwhelm the endogenous antioxidant defense system, leading to oxidative stress [23], [24]. This should associate with the abrupt and abundant O_2_ re-introduction to ischemic tissues since ROS levels of hypoxemically resuscitated animals were low and similar to those of sham group and lipid peroxidation products (MDA) and anti-oxidant reserves (GSH) were maintained. Interestingly, and perhaps paradoxically, the higher GSH levels of the Hypox-Res animals compared with sham animals, may erroneously lead to the conclusion that graded oxygenation at the early reperfusion period may amplify the antioxidant pool. Apart from the lower degree of GSH consumption, its higher level should correlate with fluid redistribution secondary to shock resuscitation that Hypox-Res animals were subjected.

As aforementioned emphasized, initial phase of ischemia and Kupffer cell activation contribute to ROS production and oxidative stress. Further, Kupffer cells activation leads to the production of proinflammatory cytokines which potentiate the hepatic I/R injury [25]. In the present study, serum TNF-α, and interleukins (IL-1β and IL-6) were significantly attenuated in the Hypox-Res compared with Normox-Res group. It may be suggested that limited cell activation occurs, ensuing less inflammatory reaction, in the case of graded re-oxygenation at the initial reperfusion period. These findings verify previous results in earlier experimental studies performed from our group, showing that hypoxemic reperfusion after hemorrhage and resuscitation in animal models limits serum TNF-α and IL-1β levels elevation when compared with normoxemic resuscitation [17], [26]. Here again the fact that peripheral blood cytokine data conduct conclusions about liver inflammatory response may raise controversy. However, it should not escape attention that liver with its great phagocytic potential may guide the inflammatory reaction when stimulated as it is the case in severe shock. In fact, in multiple organ failure with liver involvement, cytokines at the hepatic blood efflux are higher than periphery and target organs (eg lungs), a finding suggesting liver as a cytokine source that maintains and propagates organ failure [27].

In response to the exposure to inflammatory mediators such as TNF-α, IL-1α or IL-1β, neutrophils accumulate in the liver vasculature [14], [22]. These partially activated and primed neutrophils are recruited into sinusoids and post-sinusoidal or portal venules without causing tissue damage at least in the initial phase of reperfusion [28]. In view of the critical role of neutrophils in I/R injury, we used MPO as an index of the accumulation of neutrophils in the liver, because MPO activity is directly proportional to the neutrophil count and tissue injury caused by I/R [29]. Increased MPO activity was observed in both study groups at 120 min of reperfusion, however, significantly higher activity was observed in the Normox-Res group. Since the activated neutrophils are a potential source of ROS, the beneficial effect of hypoxemic reperfusion on tissue injury could be to a certain extent attributed to a reduced amount of neutrophil accumulation and activation in this early phase of reperfusion.

Increasing experimental data suggest that NO is an important component of I/R-induced tissue injury [6], [30], [31]. The role NO plays in this process is an area of active investigation and intense debate. Whether NO protects or injures, probably depends on the type of insult, the source and amount of NO production, and the cellular redox status of liver [32], [33]. For instance, endogenous NO, produced by an early and transient activation of e-NOS, protects both hepatocytes and endothelial cells against reperfusion injury in the liver [6], whereas NO by i-NOS potentiates the hepatic oxidative injury in warm I/R [33]. Supporting the latter observation, Kan et al, showed that selective i-NOS inhibition attenuated trauma-hemorrhage/resuscitation-induced hepatic injury [7]. However, others have found opposing results by showing aggravation of tissue injury during hepatic warm I/R [34].

Our results provide evidence that early reperfusion injury induces increase in e-NOS expression in liver tissue of both study groups although borderline in Hypox-Res group compared with sham. Of note, e-NOS activity differs significantly between the two groups, with the lower levels observed in the animals resuscitated under hypoxic conditions. This seems to be contradictory, considering the protective role of e-NOS [31], [35] in early I/R tissue injury, coupled with reduced hepatic damage scores (transaminases, histology) seen in Hypox-Res group compared with Normox-Res group. Low e-NOS activity in the former group could be attributed to resuscitation under hypoxic conditions, since low oxygen tension can reduce the steady-state levels of e-NOS mRNA levels of endothelial cells [36], [37].

Animal studies have shown evidence of the contribution of i-NOS on hepatic warm I/R injury [38], [39]. This enzyme is not expressed in the liver under normal conditions. It is inactive in liver ischemia, but becomes rapidly up-regulated in hepatocytes and resident hepatic macrophages upon reperfusion in response to inflammatory stimuli [8]. Collins et al demonstrated that i-NOS mRNA expression is up-regulated early, within 1 h, in hepatocytes during hemorrhagic shock [40], while Hur et al showed that it continues to rise after 5 h of reperfusion [41]. Our results are in line with these findings showing increased expression of i-NOS mRNA early, after 2 h of reperfusion. Interestingly, this was mostly detected (twelve out of sixteen livers examined) in animals resuscitated under normoxemic conditions. By contrast, only few (three out of fifteen) livers showed i-NOS gene expression in the Hypox-Res group. This finding did not differ from sham animals (Sham vs. Hypox-Res, NS). Once i-NOS is expressed, large amounts of NO are produced, which in the presence of superoxide can form peroxynitrite, a potent oxidant and protein nitrating agent. Peroxynitrite subsequently reacts with CO_2_ to produce ONOOCO_2_^−^ which decomposes to the carbonate and nitrogen dioxide radicals. Tyrosine nitration proceeds via oxidation of tyrosine to a radical intermediate which then is nitrated via radical-radical addition of NO_2_, exerting cytotoxic effects contributing to hepatic injury [42]. The detection of nitrotyrosine establishes that a marked increase in tyrosine nitration has occurred and has been widely used as a marker of peroxynitrite production. [11]. Nevertheless, protein nitration may also occur in the presence of high MPO levels as it is the case of the Normox-Res group [43]. The current study showed immunohistochemically that increased nitrotyrosine formation and marked hepatic damage were present in the Normox-Res group, indicating that normoxemic reperfusion is associated with excessive peroxynitrite formation and that the beneficial effect of hypoxemic resuscitation is at least in part, due to the prevention of peroxynitrite and MPO generation.

The histopathological data showing a significant lower Suzuki score for Hypox-Res group further support the beneficial effect of hypoxemic resuscitation on hepatocellular injury. Specifically, similarly to sham, Hypox-Res animals had reduced sinusoidal congestion, while they exhibited no hepatocyte necrosis compared with Normox-Res rabbits (p<0.05, data not shown). In accordance with the morphological observations, serum ALT was decreased at all time points, although they did not attain significance compared with the Normox-Res animals with the exception of 120 min of reperfusion.

## Materials and Methods

### Study design and setting

The study is a prospective, randomised, controlled animal study conducted in the experimental laboratory of a university intensive care unit. The protocol was approved by the Veterinary Directorate of the Prefecture of Athens according to Greek legislation in conformity with the Council Directive of the European Union. Protocol approval ID is K/3400-30/08/05.

Forty-two adult male New Zealand white rabbits of mean (±SD) body mass 3.22±0.24 kg (median = 3.1, quartiles = 3.06–3.4) fasted overnight with access to water ad libitum were induced to anesthesia with ketamine (35 mg/kg) and xylazine (5–10 mg/kg) intramuscularly.

The previously described experimental model [19] of hemorrhagic shock and resuscitation was applied with modifications. Briefly, tracheostomy was performed and mechanical ventilation (Siemens 900, Erlangen, Germany) was applied in anesthetized animals, with a PaO_2_ of 95–105 mmHg. Anesthesia was maintained with inhaled 0.5% isoflurane.

Following a 30-min stabilization period, animals were randomly assigned to sham or shock groups (first randomization). Induction to shock was conducted by continuously collecting blood from the right carotid artery allowing mean arterial pressure (MAP) to gradually decrease to 30 mmHg in a period of 30 minutes. Shock was sustained for 60 minutes at this particularly low level of MAP in order to exceed liver's increased ischemic tolerance originated from its dual perfusion blood supply; thus the potential to reach the spectrum of oxygen deprivation to attain ischemic liver injury was more probable. Animals of the sham group (sham group, n = 11) underwent all procedures of the experimental set-up with the exception of shock.

Resuscitation was started by the infusion of shed blood plus twice its volume in Ringer's lactate administered in 30 min and animals were randomly allocated into two groups (second randomization): those receiving normoxemic resuscitation (PaO_2_ = 95–105 mmHg, Normox-Res group, n = 16) by ventilation with FiO_2_ = 0.21–0.28, and those receiving hypoxemic resuscitation (PaO_2_ = 35–40 mmHg, Hypox-Res group, n = 15) by ventilation with FiO_2_ = 0.08–0.10 that begun before the onset of resuscitation. In the later group FiO_2_ was gradually increased by 0.02 every 10 minutes to achieve normoxemia by the 60 min of resuscitation [17]. Animals of all groups were followed till the 120 min of resuscitation.

### Data collection and processing

Whole blood in ethylenedinitrilotetraacetic acid (EDTA) was sampled for prompt measurement of ROS and samples of serum were stored at −80° C for subsequent measurements. At the 120 min of resuscitation liver pieces were excised from the central region of the left hepatic lobe. They were either immersed in 10% formaldehyde solution for histopathological and immunohistochemical study or stored at −80° C for subsequent measurements.

### Methods of measurement

#### Serum liver function assay

Serum ALT level was measured with a standard clinical automated analyzer (ILab 600, Instrumentation Laboratory, Shimadzu Co, Japan).

#### Molecules detecting hepatic oxidative injury

Malondialdehyde (MDA), reduced GSH, total GSH [17] and MPO activity [18] were measured in homogenized hepatic tissue as previously described.

#### Measurements of serum ROS generation and cytokines levels

The production of ROS was measured by flow cytometry using dichlorohydrofluiresceine diacetate (DCFH-DA) (Molecular Probes, Oregon, USA) as a probe as previously described [44]. The results were expressed as geometric mean of the fluorescence intensity (GMFI) for the neutrophils and monocytes of peripheral blood. Serum IL-1β, IL-6 and TNF-α protein levels were measured by Rabbit Enzyme-Linked Immunosorbent Assay (ELISA) as previously described [17].

#### Reverse transcriptase polymerase chain reaction (RT-PCR) for e-NOS, i-NOS mRNA expression GAPDH

**Figure 5:**
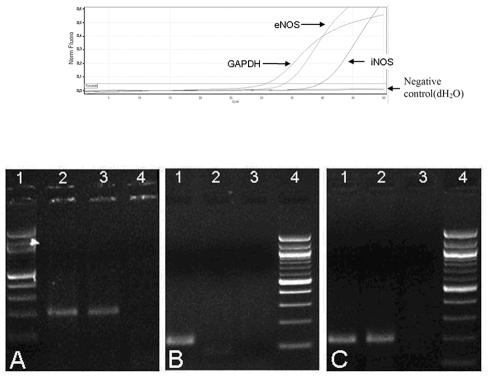
RT-PCR results for e-NOS and i-NOS. Upper panel: Real Time amplification plots of a positive for endothelial NO synthase (e-NOS) and inducible NO synthase (i-NOS) genes sample. Lower panel: Agarose gel electrophoresis (2% agarose, 80Volts, 200 mA) of RT-PCR products A: agarose gel for e-NOS (180 bp). 1. 100 bp DNA ladder 2. Sample positive for the e-Nos gene 3. Sample positive for the e-Nos gene 4. Negative control (ddH_2_O as template) B: agarose gel for i-NOS (119 bp). 1. Sample positive for the i-Nos 2. Sample negative for the i-Nos 3. Negative control (ddH_2_O as template) 4. 100 bp DNA ladder C: agarose gel for GAPDH (209 bp). 1. Sample positive for the housekeeping gene (GAPDH) 2. Sample positive for the housekeeping gene (GAPDH) 3. Negative control (ddH_2_O as template) 4. 100 bp DNA ladder.

Total RNA was extracted using TRIzol ® Reagent (Invitrogen). DNase treatment was performed using DnaseI (Invitrogen) to remove DNA traces, according to the manufacturer's instructions. Subsequently, 1 µg of RNA was subjected to reverse transcription, using M-MLV reverse transcriptase and random hexamers (Invitrogen) according to the manufacturer's instructions. The expression levels of mRNA e-NOS and i-NOS were accessed by Real Time PCR (RotorGene 6000™, Corbett Life Science). Briefly, 5 µL of cDNA were amplified with the use of the intercalating dye SYBR Green I using Platinum SYBR Green qPCR SuperMix-UDG (Invitrogen) at a final concentration 1×. Glyceraldehyde phosphate dehydrogenase (GAPDH), a constitutively expressed housekeeping gene, was also amplified under the same conditions and used to normalize reactions. Real Time PCR conditions were: Initial denaturation at 95 °C for 10 minutes, followed by 45 cycles of 95°C for 20 seconds, 57°C for 20 seconds and 72°C for 30 seconds. Relative quantification of gene expression was calculated by the 2^−ΔΔCT^ method, using one of the samples as calibrator (Figure 5, upper panel). To ensure the specificity of the reaction, the size of the PCR product for each gene was verified by 2.0% agarose gel electrophoresis (Figure 5, lower panel). Additionally, sequencing of selected positive samples was performed on the DNA ABI PRISM® 3130 using the BigDye Terminator Cycle Sequencing kit. The designed primer sequences are presented in Table 2 [45], [46], [47].

**Table 2:** Designed primer sequences.

Primer	Sequence
i-NOS-F1	CTGTGACGTCCAGCGCTACA
i-NOS-r1	GCACGGCGATGTTGATCTCT
e-NOS-F1	TGATGTGGTGTCCCTCGAAC
e-NOS-R1	ACGCTGTTGAAGCGGATTTT
GAPDHF	ACCCTCACCGCTACAACATC
GAPDHR	TCTGGCCTTCTGCTCATTCT

#### Histological examination and microscopic scoring

After fixation in 10% formaldehyde, livers were cut and processed for paraffin embedding and stained with hematoxylin-eosin. They were scored for injury by two dedicated liver pathologists blinded to the samples using the system devised by Suzuki et al [48]. Histological changes were sought in the hepatic lobule and particularly in zone III [49], the most affected area from ischemia; they were assessed semi-quantitatively for sinusoidal congestion, cytoplasmic vacuolization, and necrosis of parenchymal cell and scored on a 4-point scale (0, none; 1, slight; 2, moderate; 3, severe). The sum of the partial scores resulted in the final grading from 0 to 12. Since neutrophil infiltration is not included in Suzuki score, it was additionally assessed in lobules and perivenular areas and was graded according to the originally described system by Ishak et al [50]. The inter-observer variability was <5%.

#### Immunohistochemistry for liver nitrotyrosine detection and scoring

Tissue slides (4 µm thick), were used for immunohistochemistry using the automatic Bond Vision BioSystem (Invetech, San Diego, USA) [51]. The Bond system automates immunohistochemistry using the unique Covertile technology and Novocastra optimized reagents (Bond, Peloris and Novocastra Reagents, Newcastle, UK). Nitrotyrosine antigen was assessed with rabbit polyclonal nitrotyrosine antibody (Cell Signaling Technology, Danvers, USA). The chromogene 3,3 diaminobenzidine tetrahydrochloride was used for the staining of nitrotyrosine. The whole slide was scored semi-quantitatively for the intensity and extension of nitrotyrosine staining in the hepatic lobule and zone III in particular, on a 4-point scale (0, none; 1, slight; 2, moderate; 3, severe). The inter-observer variability was <5%.

#### Statistical analysis

Results were expressed as means ± SE or medians and interquartile range (25th and 75th percentiles) depending on the normal or non-normal distribution of values. Comparisons between groups were performed by Mann-Whitney U test with post-hoc Bonferroni analysis. Any value of p below 0.05 was considered statistically significant.
